# Knee Symptomatic Osteoarthritis, Walking Disability, NSAIDs Use and All-cause Mortality: Population-based Wuchuan Osteoarthritis Study

**DOI:** 10.1038/s41598-017-03110-3

**Published:** 2017-06-12

**Authors:** Qiang Liu, Jingbo Niu, Hu Li, Yan Ke, Rujun Li, Yuqing Zhang, Jianhao Lin

**Affiliations:** 1 Peking University People’s Hospital, Arthritis Clinic & Research Center, Beijing, 100044 China; 2 Boston University Clinical Epidemiology Research and Training Unit, the Department of Medicine at Boston Medical Center, Boston, 02118 USA

## Abstract

Knee symptomatic osteoarthritis (SxOA) was associated with all-cause mortality. Walking disability and NSAIDs use have been postulated as potential mechanisms linking knee SxOA to all-cause mortality. Data were collected on ability of walking for 1 kilometer and use of NSAIDs at baseline and death information at follow-up. Subjects with knee SxOA were identified if at least one knee had both radiographic OA and pain. We first fitted a Cox proportional hazards model to examine the relation of knee SxOA to the risk of all-cause mortality. We then used marginal structural models to decompose total effect of knee SxOA on all-cause mortality into indirect and direct effects via walking disability and use of NSAIDs, respectively. Among 1025 subjects, 99 died over 8 years of follow-up. A multivariable adjusted hazard ratio of mortality for SxOA was 1.98 (95% CI: 1.09–3.62). The indirect effect of knee SxOA on all-cause mortality through either a walking disability or NSAIDs use was 1.92 (95% CI: 0.86–4.26) and 1.45 (95% CI: 0.72–2.92), respectively. The corresponding direct effect was 1.08 (95% CI: 0.55–1.12) and 1.35 (95% CI: 0.75–2.44). In this population-based cohort study, high all-cause mortality from knee SxOA was mediated mainly through a walking disability.

## Introduction

Osteoarthritis (OA) is the most common form of joint disorder, with knee OA being a leading cause of disability among older adults globally^1–3^. Several studies have also assessed the relation of knee OA to the risk of mortality^4–9^. Although findings are inconclusive, a few recently published studies have reported that knee symptomatic OA (SxOA) was associated with a high risk of all-cause mortality^4–6^. For example, in a population-based cohort study conducted in England all-cause mortality among patients with knee or hip OA was 55% higher than that among the general population^6^. Results from the Ontario Hip and Knee Study also showed that severity of knee and/or hip OA was strongly associated with increased all-cause mortality^5^. We previously also reported that knee SxOA was associated with an approximately 2-fold increased risk of all-cause mortality among subjects in the Wuchuan Osteoarthritis Study^4^.

The underlying mechanisms linking knee SxOA to high mortality are not fully understood. Several mechanisms have been postulated to account for such an association. First, patients with knee SxOA are likely to suffer from a walking disability^10^ (i.e., impaired either gait speed^11^ or walking endurance^12^). Several studies have reported that walking disability is associated with an increased risk of all-cause mortality^6, 13^. Results from The Studies of Osteoporotic Fracture found that hip radiographic OA (ROA) was associated with increased all-cause mortality and approximately 43% were attributed to physical function involving the speed to complete a 6-meter walk^14^. Second, many knee SxOA patients took NSAIDs to treat their joint pain. Studies have shown that NSAIDs use is associated with an increased risk of cardiovascular disease and death^15^; thus NSAIDs use may be another mechanism through which patients with knee SxOA experienced a high risk of mortality^16, 17^. To our knowledge, few, if any, studies have quantitatively assessed to what extent increased all-cause mortality among patients with knee SxOA was mediated through either walking disability (i.e., the impaired walking endurance or gait speed) or use of NSAIDs.

Using data collected from the population-based longitudinal Wuchuan Osteoarthritis Study, we performed a mediation analysis to assess whether all-cause mortality observed among knee SxOA patients was mediated through either a walking disability or NSAIDs use; and, if it was, to what extent increased mortality was attributed to such mechanisms.

## Material and Methods

### Study design and Participants

Wuchuan Osteoarthritis Study is a population-based longitudinal study of natural history and risk factors for knee OA. The details of the study have been published previously^18^. Briefly, 1025 residents aged ≥50 years were recruited using door-to-door enumeration in randomly selected rural communities in Wuchuan, China between 8/2005–10/2005. Subjects completed a home interview on self-rated health status and had a hospital examination, including weight-bearing posteroanterior semiflexed view of radiographs at tibiofemoral joints (TF) and skyline view of radiographs at patellofemoral (PF) joints. A follow-up visit of study participants was conducted approximately 8 years later (11/2013). During the follow-up visit participants were queried with the same questionnaire and received the same clinical examinations as that at the baseline visit. Trained health professionals administered the survey questionnaires. At both baseline visit and follow-up, all interviewers, clinical examiners and X-ray technicians were trained under the supervision of the principal investigators of the study^4, 18^. Deaths during the follow-up was confirmed either by the interview of deceased subject’s relatives or by a search of government records. Approval was obtained from the Ethics Committee of Peking University People’s Hospital, Beijing, China (Approval Number: No. 2012–040). All methods were performed in accordance with the relevant guidelines and regulations. Written informed consent was obtained by all study participants.

### Assessment of Exposure, Mediators and Outcome

Knee ROA was assessed using weight-bearing posteroanterior semiflexed view of radiographs at TF and skyline view of radiographs at PF joints. Each knee was evaluated for the presence of radiographic TF ROA based on Kellgren/Lawrence (K/L) criteria^19^. ROA at PF joint was identified if osteophyte grade is ≥2 or if JSN grade is ≥2 (on a 0–3 scale) with concurrent grade 1 osteophyte in the patellofemoral joint. The weighted kappa on K/L grade for inter-rater reliability was 0.80 (95% confidence interval (CI): 0.72–0.88) and the intra-rater reliability was 0.92 (95% CI: 0.86–0.99)^18^. Knee pain symptoms were queried using the following question “Did knee pain occur on most days in the past month?” at the baseline home interview. We defined a knee as having ROA if either K/L score at the TF joint ≥2 or presence of PF ROA. Knee SxOA was recorded if both pain and ROA were present at the same knee.

At the baseline examination, all subjects were asked if they were able to perform various daily activities, including current ability of walking two Li (the native expression of one kilometer) “with no difficulty”, “some difficulty”, or “very difficult/unable to do”. In the current analyses, we defined a subject as having a walking disability if they responded to the question above with “very difficult or unable to do”. During the home interview, subjects were asked whether they have taken in the past 12 months or are currently taking any medications, including NSAIDs, to relieve their knee pain. The use of NSAIDs was asked in both chemical and trade name to reduce misclassifications, including Ibuprofen, Diclofenac, Indomethacin, Loxoprofen Sodium, Flurbiprofen, Naproxen, Diflunisal, Fenoprofen, Ketoprofen, MefenamicAcid, Nabumetone, Oxaprozin, Salsalate, Tolmetin, Etodolac, Ketorolac, Meloxicam, Piroxicam, Sulindac and Celebrex.

Information on death during the follow-up period was obtained either by interviewing relatives of the deceased subjects or by searching documents from the Birth and Death Registry of the local community office.

### Assessment of Confounders

At the baseline visit, data were collected on socio-demographic factors, including age, sex, education level and family income. Occupational physical activity was assessed based on the longest job they took during their lifetime, and levels of occupational physical activity were grouped into four categories: sedentary, light physical activity, moderate physical activity and heavy physical activity. Subjects were asked whether they have currently suffered from each specific disease, including hypertension, diabetes, heart diseases, chronic lung diseases, renal disorders and malignant tumor. Height was measured twice for each subject using a wall-mounted stadiometer and the average of these two measures was used in the analysis. Weight was assessed using a balance beam scale with a precision to 0.1 kg. Body mass index (BMI) was calculated as weight in kilograms divided by height in meters squared.

### Statistical Analyses

Person-years of follow-up were computed as the amount of time from the date that the radiographs were obtained to the date of the following events: death, the last date of contact for those lost to follow-up, or follow-up visit in 2013, whichever comes first. All-cause mortality according to knee SxOA was calculated by dividing the number of death by the number of person-years of follow-up. We fitted a Cox proportional hazards model, using age as the time scale, to examine the relation of knee SxOA to the risk of all-cause mortality adjusting for baseline age, sex, BMI, education level, income, level of occupational physical activity, and comorbidities, including hypertension, diabetes, heart diseases, chronic lung diseases, renal disorders and malignant tumor^20, 21^. Age and BMI were adjusted as continuous variables; sex, education level (middle school level or higher vs. primary school of illiteracy), income (≥4000 Yuan vs. <4000 Yuan), level of occupational physical activity (heavy vs. other), and comorbidities (no vs. 1 or more).

**Figure 1:**
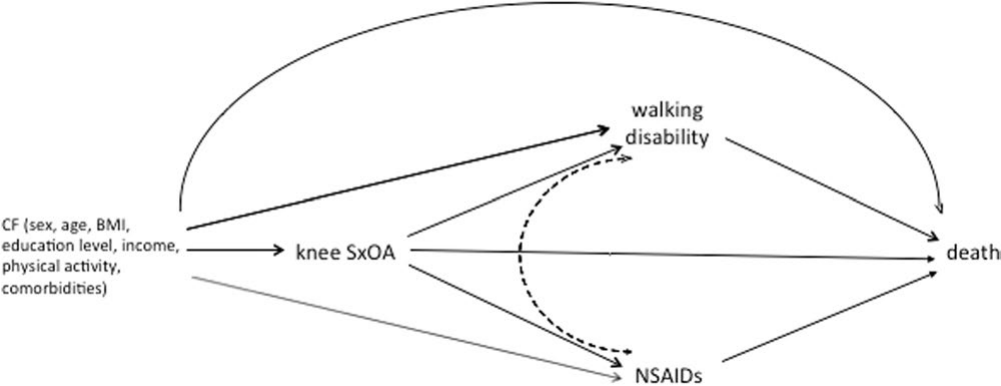
A directed acyclic graph to describe the hypotheses: we decomposed the total effect of knee symptomatic osteoarthritis (SxOA) on all-cause mortality into two components: 1) the indirect effect (or mediated effect), i.e., the effect of knee SxOA on all-cause mortality mediated through either a walking disability (knee SxOA → walking disability → death) or Nonsteroidal antiinflammatory drugs (NSAIDs) use (knee SxOA → NSAIDs → death), and 2) the direct effect, i.e., the effect of knee SxOA on all-cause mortality (knee SxOA → death) that was not through either a walking disability or use of NSAIDS. The dotted line between walking disability and NSAIDs indicated that the time sequence was uncertain. CF: confounders.

To assess whether the association between knee SxOA and all-cause mortality is mediated through its effect on walking disability, we used marginal structural model to decompose the total effect of knee SxOA on all-cause mortality into two components^22^ (Fig. 1): 1) the indirect effect (or mediated effect), i.e., the effect of knee SxOA on all-cause mortality mediated through a walking disability, and 2) the direct effect, i.e., the effect of knee SxOA on all-cause mortality that was not through a walking disability. We used the same approach to assess mediation effect of NSAIDs use. Use of NSAIDs was analyzed as dichotomized variable. All statistical analyses were performed using SAS 9.3 (SAS Institute Inc., Cary, NC, USA).

### Data availability

The datasets analysed during the current study are not publicly available due to conservation regulations but are available from the corresponding author on reasonable request.

## Results

At baseline of the Wuchuan Osteoarthritis Study, a total of 1165 individuals aged 50 years and older were identified and 1030 (91%) consented to participate in the study. Five subjects without knee radiographs were excluded from analysis. More details were provided in the previously published work^4, 18^. 1025 participants were followed until the end of 2013. Of them, 63 (6.1%) had knee SxOA and 99 subjects died during the followup period.

Baseline characteristics of subjects are shown in Table 1. Participants with knee SxOA were more likely to be women, older, had lower annual income, and higher BMI than those without knee SxOA. No apparent difference was observed in levels of education and occupational physical activity according to status of knee SxOA. The prevalence of walking disability and NSAIDs use was higher among subjects with knee SxOA than those without it.

**Table 1:** Baseline Characteristics of Participants, Wuchuan Osteoarthritis Study, China.

Characteristics	Symptomatic Knee OA		
	Yes (n = 63)	No (n = 962)	P-value*
Women (%)	71.4	49.4	<0.001
Age (years, mean ± SD)	62.2 ± 8.6	56.0 ± 7.8	<.001
Education (%)		0.622	
Elementary School	66.7	69.1	
Middle School	30.2	25.5	
High School and more	3.2	5.4	
Annual income ≥3000 yuan	39.7	55.8	0.003
BMI (kg/m^2^, mean ± SD)	23.9 ± 3.7	22.3 ± 3.2	0.002
Levels of Physical Activity (%)		0.111	
Light	0.0	0.7	
Moderate	15.9	7.7	
Heavy	84.1	91.6	
Walking disability (%)	22.2	5.0	<.001
Use of NSAIDs (%)	84.1	38.9	<.001

^*^P-values were from t-test for continuous variables and from Chi-square test for categorical variables.

As shown in Table 2, while subjects with either walking disability or use of NSAIDs were associated with increased risk of all-cause mortality, neither the effect estimate reached statistical significance. The multivariable adjusted hazard ratios of all-cause mortality were 1.32 (95% CI: 0.74–2.36) for walking disability and 1.45 (95% CI: 0.93–21.26) for use of NSAIDs, respectively.

**Table 2:** Walking Disability, Use of NSAIDs and All-Cause Mortality.

		No.Deaths/No.Subjects	Crude HR (95% CI)	Adjusted HR (95% CI)^*^
Walking Disability	No	76/924	1.0	1.0
	Yes	23/101	1.39 (0.84–2.29)	1.32 (0.74–2.36)
NSAIDs Use	No	48/598	1.0	1.0
	Yes	51/427	1.26 (0.84–1.88)	1.45 (0.93–2.26)

*Adjusting for age, sex, BMI, education, income level, level of daily physical activity, comorbidities, and knee SxOA.

As shown in Table 3, all-cause mortality rate was higher among subjects with knee SxOA (32.6/1000 person-years) than those without the disease (10.9/1000 person-years). Compared with those without knee SxOA, the multivariable adjusted hazard ratio (HR) of all-cause mortality for knee SxOA (i.e., the total effect of knee SxOA) was 1.98 (95% CI: 1.09–3.62). The indirect effect (i.e., mediated effect) of knee SxOA on all-cause mortality via a walking disability was 1.92 (95% CI: 0.86–4.26), almost the same as its total effect. However, the direct effect of knee SxOA on all-cause mortality not through a walking disability was null (HR = 1.08, 95% CI: 0.55–2.12), suggesting that the effect of knee SxOA on all-cause mortality was almost entirely (i.e., 97.0%) mediated through a walking disability. In contrast, the indirect effect of knee SxOA on all-cause mortality though NSAIDs use was 1.51(95% CI: 0.71–2.92) and the direct effect was 1.42 (95% CI: 0.79–2.41), indicating that effect of knee SxOA on all-cause mortality was only partly (76.2%) mediated through NSAIDs use. When we excluded 11 subjects whose vital status could not be confirmed, the mediation effect of disability of walking one kilometer or NSAIDs use did not change materially.

**Table 3:** Total, Direct and Indirect Effect of Knee SxOA on All-Cause Mortality Mediated through Walking Disability and Use of NSAIDs.

SxOA Status	No.Deaths/No.Subjects	Total Effect HR (95% CI)	Indirect Effect HR (95% CI)	Mediation Effect (%)	Direct Effect
			Via Walking Disability		
No	84/962	1.0	1.0		1.0
Yes	15/63	1.98 (1.09–3.62)	1.92 (0.86–4.26)	97.0	1.08 (0.55–2.12)
			Via NSAIDs Use		
No	84/962	1.0	1.0		1.0
Yes	15/63	1.98 (1.09–3.62)	1.51 (0.72–2.92)	76.2	1.42 (0.79–2.41)

*Adjusting for age, sex, BMI, education, income level, level of daily physical activity, and comorbidities.

## Discussion

In this population-based cohort study conducted among residents living in rural areas of China, knee SxOA was associated with a high all-cause mortality. The effect of knee SxOA on all-cause mortality was mainly mediated through its effect on walking disability (i.e., unable to walk for at least 1 kilometer in this study). In addition, NSAIDs use also partly mediated the effect of knee SxOA on all-cause mortality.

The lifetime risk of knee SxOA is estimated to be approximately 45% among US population^23^. Previous studies have found that knee SxOA is more common among Chinese than that among Caucasians, especially among people living in the rural areas of China where socioeconomic development and health care system are not as good as that in the urban areas^18, 19^. Knee SxOA constitutes a tremendous disease burden owing to pain, functional limitation and physical disability^24, 25^.

The potential mechanisms linking OA to all-cause mortality have not been well elucidated. To date, a few mechanisms have been postulated to account for such an association. First, previous studies have shown that patients with knee SxOA are more likely to suffer from walking disability^26–28^. In the current study, 22.2% of patients with knee SxOA had walking disability (i.e., had difficulty in or were unable to walk for 1 kilometer). Previous studies have shown that walking disability is a strong predictor for all-cause mortality^6, 13^. Thus, increased all-cause mortality among patients with knee SxOA might be mediated via walking disability. Second, numerous studies have reported that NSAIDs use is associated with an increased risk of cardiovascular disease, gastrointestinal bleeding, and mortality^15, 29, 30^. Considering many patients with knee SxOA take NSAIDs to relieve their joint pain and often use NSAIDs for a long period of time; thus an increased risk of all-cause mortality observed among patients with knee SxOA could be partially mediated via NSAIDs use. In our study 84.1% of patients with knee SxOA took NSAIDS for treatment of their knee pain, whereas only 38.9% subjects without knee SxOA took NSAIDs. The NSIADs use among participants with knee SxOA in our study is common. Owing to its high cost and lack of qualified orthopedic surgeons, the total knee replacement therapy is not widely available for patients with the end stage of knee OA in China, especially for those living in the rural areas. Therefore, NSAIDs use becomes the most common treatment regimen for pain relief and symptoms improvement because of its low cost and easy access. On the other hand NSADIS use has been reported to be associated with increased risk of gastro-intestinal bleeding and cardiovascular diseases. However, we did not collect such information in our study. Considering the high prevalence of knee SxOA and NSAIDs use, future studies should be conducted among subjects with knee SxOA and to assess whether risks of these diseases as well as cause-specific mortality are indeed increasing in this population. Nevertheless, other non-pharmacological therapies, such as appropriate physical activity/exercise, braces or insole, and walking supports, could be considered as alternatives to reduce the knee symptoms.

While these hypotheses point to plausible causal pathways between knee SxOA and all-cause mortality, few studies have formally tested these hypotheses and quantitatively estimated to what extent each of these mechanisms account for effect of knee SxOA on all-cause mortality. Recently, Barbour *et al*. found that radiographic hip OA was associated with increased all-cause mortality among participants in The Studies of Osteoporotic Fracture, and estimated that approximately 40% of such an increased mortality was mediated through function impairment. Our study also showed that walking disability was a strong mediator for the association between knee SxOA and all-cause mortality, and provided further support for the validity of these findings.

Ability to walk, especially walking long distances (i.e., walking endurance), has been considered as a “vital sign” of overall health and wellbeing. Results from the Health, Aging, and Body Composition study found that subjects who were unable to complete walking 400 meters had a 38% increased all-cause mortality than those who were able to complete the task. Furthermore, among those who were able to complete the 400-meter walking test, subjects in the poorest quartile of functional capacity (i.e., walking time >362 seconds) had more than a 3-fold (HR = 3.23, 95% CI: 2.11–4.94) increased risk of death than those in the best quartile (walk time <290 seconds). In the current study we found that almost all increased risk of death observed among patients with knee SxOA was mediated through walking disability. This finding is not surprising because walking ability, particularly walking endurance, is more crucial to people living in rural areas of China than those living in the United States given that most daily-living activities cannot be performed properly owing to limited social and community supporting services. Elucidation of potential biological mechanisms through which knee SxOA causes all-cause mortality will not only shed light on our understanding the disease pathophysiology, but also guide us to develop more efficient preventive and treatment strategies. Our study, in collaboration with other studies, call for further studies to evaluate the effect of improvement of physical function, such as walking endurance, on all-cause mortality among subjects with knee SxOA^31^.

Several characteristics of our study are noteworthy. First, our study is a general population-based cohort study. Participants were followed over a long period of time and the rate of lost to follow-up was low; thus we were able to assess death accurately. Second, we were able to adjust for most important potential confounders and our results were similar to that published recently, suggesting the findings were robust. Third, when we excluded subjects with extreme stabilized weights (i.e., mean ± 3 standard deviation of stabilized weights), the results did not change materially. In fact, the magnitude of indirect effect of knee SxOA on all-cause mortality via walking disability increased slightly albeit with wide confidence intervals due to relatively small sample size in the current study. Finally, our study found that knee SxOA was associated with an increased risk of all-cause mortality among the residents living in the rural areas, and such an effect may be mediated mainly through a walking disability. Considering that a large proportion of Chinese people live in the rural areas, and prevalence of knee SxOA is much higher in the rural areas than that in the urban areas^32^; thus development of appropriate preventive and treatment strategies to reduce the risk of knee SxOA and the occurrence of walking disability should be the priority of OA research in China.

Our study has some limitations as well. First, the total number of subjects in the current study is relatively small, especially the number of subjects with knee SxOA. Although the total effect of knee SxOA on the all-cause of mortality was statistically significant, the estimates of direct and indirect effect had wide confidence intervals, reflecting the uncertainty of the results. Second, smoking, stroke and depression were not assessed in the current study and we were unable to control for its potential confounding effect. Third, in addition to possible misclassification, we only collected the information of NSAIDs use at baseline and did not assess the frequency, duration and type of these medications; thus, we were unable to assess different categories of NSAIDs use that may mediate the association between knee SxOA and all-cause mortality. Furthermore, data on the NSAID use and walking disability were collected once at the same time; thus we were unable to appropriately assess any interplay between these two potential mediators in our analysis. In addition, information on knee SxOA walking disability and NSAIDs use was collected at baseline; thus we can’t be sure that knee SxOA definitely occurred before the occurrence of walking disability and NSAIDs use. Nevertheless, many knee SxOA patients took NSAIDs to control their knee pain and no study has reported that NSAIDs use may increase the risk of knee SxOA, and studies have reported that knee SxOA is a major risk factor for limitation and disability of lower extremity tasks, such as walking and stair climbing^3^. Thus, we postulate that walking disability and NSAIDs use occurred after subjects developed knee SxOA and may likely be the potential mediators between knee SxOA and all-cause mortality. Forth, we only collected potential confounders at baseline, including history of fracture and knee injury. These two factors can either serve as the potential confounders or mediators between knee SxOA and all-cause mortality. However, when we added them in the model, it does not change either total or direct or indirect effects materially. Fifth, we did not collect data on specific causes of death in this cohort. Nevertheless, all-cause mortality is critically important in its own right, as it represents the overall net health outcome of various risks associated with knee SxOA^31^. Finally, this study was conducted among residents in the rural areas in China where health care system is underdeveloped and living standard is relatively low, our results may not be generalizable to the residents living in urban areas in China or to people living in other countries.

In conclusion, this general population-based cohort study shows that the knee SxOA is associated with a higher risk of all-cause mortality, and such an association may be mediated mainly through its effect on the capacity of walking endurance.
